# Chronic Cigarette Smoke Extract Exposure Promotes Genomic and Phenotypic Reprogramming in Lung Cancer Cells

**DOI:** 10.1111/1759-7714.70392

**Published:** 2026-10-09

**Authors:** Hyun Soo Kim, Se‐Eun Choi, In Kyoung Kim, Seulbee Lee, Seo‐Hee You, Rachel Hyunkyung Kim, Chang Dong Yeo, Jin‐A Kim, Seok Chung, Sang Haak Lee, Semin Lee, Hye Joung Kim

**Affiliations:** ^1^ School of Mechanical Engineering Korea University Seoul Korea; ^2^ Department of Biomedical Engineering, College of Information and Biotechnology Ulsan National Institute of Science and Technology Ulsan Korea; ^3^ Division of Pulmonary, Department of Internal Medicine, College of Medicine The Catholic University of Korea Seoul Republic of Korea; ^4^ Cancer Research Institute, College of Medicine The Catholic University of Korea Seoul Republic of Korea; ^5^ KU‐KIST Graduate School of Converging Science and Technology Korea University Seoul Korea; ^6^ Institute of Chemical Engineering Convergence System Korea University Seoul Korea

**Keywords:** chronic exposure, cigarette smoke extract, genomic instability, homologous recombination, lung cancer progression, mutational signatures

## Abstract

**Background:**

Cigarette smoke contains numerous toxic compounds that can induce oxidative stress and DNA damage. While smoking is a well‐established driver of lung cancer initiation, the biological consequences of continued exposure after tumor establishment remain poorly understood. This study investigated the genomic and phenotypic effects of chronic cigarette smoke exposure in established lung cancer cells.

**Methods:**

Human lung adenocarcinoma A549 cells were chronically exposed to cigarette smoke extract to model sustained exposure. Cellular proliferation and migration assays were performed to evaluate phenotypic changes. Transcriptomic profiling was used to examine alterations in gene expression related to cell cycle regulation and DNA repair pathways. Whole‐genome sequencing was conducted to identify mutational signatures associated with genomic instability. Tumor growth and histological features were further evaluated using xenograft models.

**Results:**

Chronic cigarette smoke extract exposure increased the clonogenic capacity and migratory behavior of A549 cells and induced phenotypic features consistent with partial epithelial–mesenchymal transition. Transcriptomic analysis revealed disruption of cell cycle regulation and reduced expression of homologous recombination DNA repair genes, including RAD51. Whole‐genome sequencing identified mutational signatures associated with oxidative damage, mismatch repair deficiency, and homologous recombination defects. These alterations were accompanied by increased tumor growth and cellular atypia in xenograft models.

**Conclusions:**

Chronic cigarette smoke exposure may enhance genomic instability and promote aggressive phenotypes in established lung cancer cells, suggesting that continued smoking after cancer development may contribute to tumor progression.

## Graphical abstract

Chronic cigarette smoke extract exposure promoted genomic and phenotypic reprogramming in established lung cancer cells. Clonogenicity, migration, and tumor growth increased, accompanied by changes consistent with partial epithelial–mesenchymal transition. Transcriptomic analysis and whole‐genome sequencing revealed activation of inflammatory signaling, altered DNA repair programs, and smoking‐associated mutational signatures.

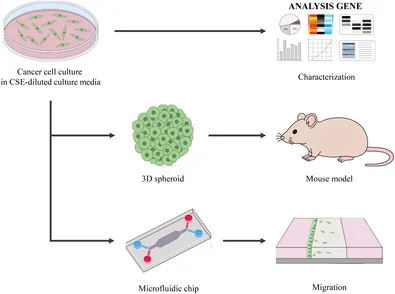

## 1 Introduction

Lung cancer remains the leading cause of cancer‐related mortality worldwide, with cigarette smoking recognized as its most significant risk factor [1, 2]. Cigarette smoke is a complex mixture containing thousands of chemical constituents, including reactive oxygen species, polycyclic aromatic hydrocarbons, and nitrosamines, many of which possess mutagenic and carcinogenic properties [3, 4]. Exposure to cigarette smoke induces oxidative DNA damage, DNA adduct formation, and disruption of cellular DNA repair pathways [3, 5, 6].

Clinical studies have demonstrated that continued smoking after lung cancer diagnosis is associated with worse survival and poorer clinical outcomes [7, 8, 9, 10, 11]. However, the biological mechanisms underlying these adverse outcomes remain incompletely understood. Many prior in vitro studies have investigated acute or relatively limited cigarette smoke extract (CSE) exposure and focused on early carcinogenic responses, such as DNA damage, calcium signaling, and EMT‐related phenotypic changes [12, 13, 14]. Even when more prolonged phenotypic effects have been explored, research has generally remained focused on selected endpoints such as EMT or drug‐response changes rather than on the cumulative transcriptomic and genomic consequences of sustained exposure in already established lung cancer cells [15, 16]. Accordingly, identifying whether chronic exposure to cigarette smoke further promotes genomic instability and aggressive tumor phenotypes in established lung cancers remains necessary, particularly through studies integrating transcriptomic and genomic analyses.

Genomic instability is a hallmark of cancer and plays a central role in tumor evolution, intratumoral heterogeneity, and therapeutic resistance [17]. Defects in key DNA repair pathways, including homologous recombination and mismatch repair, contribute to the accumulation of somatic mutations and the development of aggressive tumor phenotypes [18, 19]. In lung cancer, smoking‐associated mutational processes have been strongly linked to elevated mutational burden and characteristic oxidative damage signatures [20, 21, 22, 23, 24]. Nevertheless, whether chronic toxic exposure to cigarette smoke further exacerbates genomic instability and promotes aggressive phenotypes in already transformed lung cancer cells remains uncertain.

In this study, we investigated the genomic and phenotypic consequences of chronic CSE exposure in lung cancer cells. To address this question, we established a chronic CSE exposure model using A549 lung cancer cells and evaluated its biological effects across multiple experimental platforms. By integrating phenotypic assays with transcriptomic profiling and whole‐genome sequencing (WGS), we aimed to characterize how sustained toxic stress influences genomic stability and tumor cell behavior. Furthermore, using three‐dimensional spheroid cultures and xenograft models, we assessed whether chronic CSE exposure promotes aggressive tumor phenotypes associated with smoking‐related lung cancer. Collectively, this study provides a multi‐platform framework for investigating the genomic and phenotypic consequences of chronic cigarette smoke exposure in established lung cancer cells.

## 2 Materials and Methods

### 2.1 Preparation and Chemical Characterization of CSE

CSE was prepared as described in a previous study [25], with minor modifications. Briefly, smoke from 20 commercial cigarettes (Korea Tomorrow & Global Corp., Daejeon, Republic of Korea) was bubbled into 20 mL of phosphate‐buffered saline using a vacuum pump‐based bottle system. The resulting solution was defined as 100% CSE and filtered through a 0.22‐μm membrane to remove insoluble particles.

Prior to the cell treatment experiments, compositional analysis was performed on the same CSE batch subsequently used throughout this study using two independent and complementary analytical methods. HPLC analysis was conducted by the Korea Research Institute of Bioscience (Report No. 2020KRIBS0434.002) using an authentic nicotine standard for retention‐time comparison. GC–MS library analysis was performed by the Korea Polymer Testing & Research Institute (Koptri; Report No. Koptri‐20‐07‐09873‐1). Compounds were tentatively assigned by comparison with the GC–MS library, and the reverse match factor and relative chromatographic peak area were recorded. Detailed analytical results are provided in Table S1.

### 2.2 Cell Culture and CSE Treatment

A549 human alveolar adenocarcinoma cells (American Type Culture Collection, Manassas, VA, USA) were maintained in RPMI‐1640 medium (Gibco, Grand Island, NY, USA) supplemented with 10% fetal bovine serum (FBS) and 1% penicillin–streptomycin at 37°C in a humidified incubator containing 5% CO_2_. CSE treatment was performed as described in a previous study [25]. To establish a chronic CSE exposure model, A549 cells were subjected to repeated cycles of CSE treatment. Briefly, cells were seeded and allowed to attach for 24 h, then treated with 1.5% CSE for 48 h without medium replacement. Cells were subcultured at the end of each 3‐day cycle, and the cycle was repeated once every 3 days for a total of nine cycles over approximately 4 weeks. This repeated exposure strategy was used to mimic sustained toxic stress conditions associated with persistent smoking. CSE‐exposed A549 cells are hereafter referred to as A549 CSE.

### 2.3 Cell Viability and Colony Formation Assays

Cell viability was assessed using 3‐(4,5‐dimethylthiazol‐2‐yl)‐2,5‐diphenyltetrazolium bromide (MTT) assay (Sigma‐Aldrich, St. Louis, MO, USA). Cells were incubated with MTT solution for 4 h, after which formazan crystals were dissolved in dimethyl sulfoxide, and absorbance was measured at 570 nm using a microplate reader (Infinite M200 Pro; Tecan, Switzerland). For colony formation assays, cells were seeded at a density of 500 cells per well in 6‐well plates and cultured for 14 days at 37°C in a humidified atmosphere with 5% CO_2_, with the culture medium replaced every 3–4 days, as previously described [26]. Colonies were fixed with 4% paraformaldehyde for 15 min and stained with Wright–Giemsa solution (Cell Biolabs, San Diego, CA, USA). Colonies containing ≥ 40 cells were counted under an inverted phase‐contrast microscope (Primovert, Carl Zeiss, Jena, Germany) at 200× magnification, and colony number and area were quantified using ImageJ software (NIH, Bethesda, MD, USA). All assays were performed in triplicate and repeated in three independent experiments.

### 2.4 Scratch Wound and Invasion Assays

For scratch wound assays, a sterile 200‐μL pipette tip was used to create a linear wound approximately 1 mm in width in confluent cell monolayers. Images were captured immediately after scratching (0 h) and again after 24 h. Wound areas were measured from the images at both time points for each of three independent biological replicates. Percent wound closure was calculated separately for each replicate using the following formula: 100 × (1 − Area24h/Area0h). Statistical comparisons between the A549 and A549 CSE groups were performed using Welch's unpaired *t*‐test without assuming equal variances.

For invasion assays, Matrigel‐coated (2.5 mg/mL) Transwell chambers (Corning, NY, USA) were used. Serum‐free medium was added to the upper chamber, while medium containing 10% FBS was placed in the lower chamber as a chemoattractant. After 24 h, non‐invading cells were removed, and invading cells were fixed, stained with crystal violet, and counted in four randomly selected microscopic fields.

### 2.5 Quantitative Real‐Time Polymerase Chain Reaction (qRT–PCR)

Total RNA was extracted using TRIzol reagent (Invitrogen, Carlsbad, CA, USA) and reverse‐transcribed using a cDNA synthesis kit (Bio‐Rad Laboratories, Hercules, CA, USA). qRT–PCR was performed using TOPreal SYBR Green qPCR PreMIX (Enzynomics, Daejeon, Republic of Korea). *β‐actin* was used as an internal control. Relative gene expression levels were calculated using the 2^−ΔΔCt^ method. Primer sequences are provided in Table 1.

**TABLE 1:** Primer sequences for quantitative real‐time PCR.

Gene (human)	Forward primer (5′–3′)	Reverse primer (5′–3′)
E‐cadherin	CGGGAATGCAGTTGAGGATC	AGGATGGTGTAAGCGATGGC
N‐cadherin	ATTGGACCATCACTCGGCTTA	CACACTGGCAAACCTTCACG
Vimentin	GGACCAGCTAACCAACGACA	AAGGTCAAGACGTGCCAGAG
β‐catenin	GAGCCTGCCATCTGTGCTCT	ACGCAAAGGTGCATGATTTG
Snail	TTTACCTTCCAGCAGCCCTA	CCCACTGTCCTCATCTGATA
NOTCH1	GGTGAACTGCTCTGAGGAGATC	GGATTGCAGTCGTCCACGTTGA
TRPV4	TCACTCTCACCGCCTACTACCA	CCCAGTGAAGAGCGTAATGACC
TGFβR3	TGGAGTCTCCTCTGAATGGCTG	CCATTATCACCTGACTCCAGATC
CCL5	CCTGCTGCTTTGCCTACATTGC	ACACACTTGGCGGTTCTTTCGG
RAD51	TCTCTGGCAGTGATGTCCTGGA	TAAAGGGCGGTGGCACTGTCTA
CDK2	ATGGATGCCTCTGCTCTCACTG	CCCGATGAGAATGGCAGAAAGC
β‐actin	GATGCAGAAGGAGATCACTGC	CGATCCACACGGAGTACTTG

### 2.6 Western Blotting

Proteins were extracted using radioimmunoprecipitation assay buffer and quantified using a bicinchoninic acid protein assay kit (Thermo Fisher Scientific, Rockford, IL, USA). Equal amounts of protein were separated by 8%–12% sodium dodecyl sulfate–polyacrylamide gel electrophoresis and transferred to polyvinylidene difluoride membranes. Membranes were blocked with 5% skim milk in Tris‐buffered saline with Tween 20 and incubated overnight at 4°C with primary antibodies against E‐cadherin, N‐cadherin, Vimentin, β‐catenin, Snail, and β‐actin (Cell Signaling Technology, Danvers, MA, USA). Immunoreactive bands were visualized using an ImageQuant LAS 500 imaging system (GE Healthcare, Uppsala, Sweden) and quantified using ImageJ software.

### 2.7 Three‐Dimensional Spheroid Formation

To generate three‐dimensional spheroids, C‐well (INCYTO, Cheonan, Republic of Korea, ISF‐361‐5) was cut to cover approximately one‐half of the bottom surface of a single well of a 6‐well plate and fitted into the well. The C‐well was coated with 1% Pluronic F‐127 (Sigma‐Aldrich, P2443) and incubated for 1 h at 37°C and washed three times with PBS. A549 cells were trypsinized and counted. Based on a target density of 2000 cells per C‐well microwell, twice the calculated number of cells was suspended in 4 mL of complete culture medium and seeded into the 6‐well plate containing the C‐well. The plate was centrifuged at 2000 rpm for 3 min, after which the medium containing unattached cells was aspirated. The culture medium was replaced once on Day 2, and the tumor spheroids were used for experiments on Day 3.

### 2.8 Ectopic Mouse Model

BALB/c nude mice (5–6 weeks old, *n* = 5 per group) were subcutaneously injected in the dorsal flank with spheroids generated from either parental A549 or A549 CSE cells, suspended in 100 μL of serum‐free medium mixed with 100 μL of Matrigel. Control animals received serum‐free medium only. Mice were housed under controlled conditions (23°C ± 2°C, 55% ± 5% humidity, 12‐h light/dark cycle). After 4 weeks, mice were anesthetized with Avertin (25 μL/g body weight), tumors were excised, and tissues were processed for hematoxylin and eosin staining, as well as RNA and protein extraction. Tumor volume was calculated using the following formula:

(1) $$V=\left(4/3\right)\pi \times \left(L/2\right)\times \left(W/2\right)\times \left(H/2\right),$$

where *L*, *W*, and *H* represent length, width, and height, respectively.

All animal experiments were approved by the Institutional Animal Care and Use Committee of the Catholic University of Korea (CUMC‐2021‐0292‐06).

### 2.9 Immunohistochemical Staining Image Analysis

Immunohistochemical staining image was quantified using ImageJ software (National Institutes of Health, Bethesda, MD, USA). For each tumor section, the positively stained area was measured within the analyzed tumor tissue region. The percentage of positive staining area was calculated by dividing the positively stained area by the total analyzed tissue area and multiplying by 100, as follows: positive staining area (%) = (positively stained area/total analyzed tissue area) × 100.

### 2.10 Fabrication of Wafer and Microfluidic Chip

Microfluidic chips were fabricated as previously described [27, 28]. Briefly, silicone elastomer base and curing agent (SYLGARD 184 A/B, Dow, Midland, MI, USA) were mixed at a 10:1 ratio to prepare polydimethylsiloxane (PDMS). The mixture was poured onto patterned SU‐8 wafers. Air bubbles were removed using a vacuum chamber, and PDMS was cured at 80°C for at least 2 h. After curing, PDMS chips were detached from the wafer, and medium reservoirs and gel ports were created using biopsy punches (Ginormous Lab, Suwon, Korea). The chips were sterilized at 120°C for 15 min. The PDMS chips were bonded to cover glasses (Marienfeld, Lauda‐Königshofen, Germany) using oxygen plasma treatment. The bonded chips were coated with 1 mg/mL poly‐D‐lysine solution and incubated for 1 h at 37°C. Chips were then rinsed three times with distilled water to remove residual coating solution. After the final rinse, chips were dried overnight at 80°C to restore hydrophobicity.

### 2.11 Chip Preparation and Migration Assay

Type I collagen was prepared at a final concentration of 2 mg/mL by mixing collagen type I (Corning, 354 236), phenol red‐containing 10× PBS (Gibco, 14080055), NaOH (Sigma‐Aldrich, S2770), and distilled water; the pH was adjusted to neutral. The collagen solution was injected into the central gel channel and allowed to polymerize at 37°C for 30 min in a humid chamber. Both medium channels were then filled with complete culture medium, and the chips were incubated overnight in the incubator. The next day, medium was aspirated from the reservoirs of both medium channels. Tumor cells (1 × 10^6^ cells/mL, 50 μL) were introduced into one medium channel and allowed to attach for 30 min with the chip tilted at 90°. After cell attachment, medium was aspirated from the reservoir of the cell‐seeded channel, and both medium channels were refilled with fresh medium. The medium was replaced every 24 h. To quantify cell migration, the leading edge of the cell population on Day 1 was designated as the baseline. On Day 7, migration distance was defined as the maximum straight‐line distance from this baseline to the cell that had migrated farthest. Distances were measured using ImageJ software (National Institutes of Health, Bethesda, MD, USA).

### 2.12 RNA Sequencing (RNA‐Seq) and Data Analysis

RNA‐seq reads were quality‐checked using FastQC [29] (v0.11.9; Babraham Bioinformatics, Cambridge, UK), aligned to GRCh38 reference genome (GENCODE v38) [30] using STAR (v2.7.9a) [31], and quantified using RSEM (v1.3.2) [32]. Lowly expressed genes (total count ≤ 10) were excluded. Batch effects were corrected using ComBat‐seq [33], and differential expression analysis was performed using DESeq2 (v1.34.0) [34] (*p*_adj_ < 0.05, |log_2_[Fold change]| > 1). Normalized expression levels of *KRT4*, *RAD51*, *CDK2*, *TGFβR3*, and *CCL5* were visualized using ggplot2 (v3.5.1) [35]. Functional enrichment analysis was conducted using Enrichr [36].

### 2.13 Mutational Signature Profiling

WGS reads were aligned to the GRCh38/hg38 reference genome (Broad Institute GATK Resource Bundle) using BWA‐MEM (v0.7.17) [37] (Li H. 2013. arXiv:1303.3997v2 [q‐bio.GN]). Duplicate reads were marked with Picard (v2.9.0) (Broad Institute, GitHub Repository, RRID: SCR_006525, https://broadinstitute.github.io/picard/). Base quality score recalibration and somatic variant calling were performed using GATK (v4.2.6.0) [38, 39]. Somatic mutations were identified using Mutect2 and filtered with FilterMutectCalls following GATK Best Practices. Mutational signature assignment was performed using SigProfilerAssignment (v0.1.5) [40].

### 2.14 Statistical Analysis

Unless otherwise indicated, in vitro experiments were performed at least three independent times. Data are presented as the mean ± standard deviation (SD). For comparisons between two groups, an unpaired two‐tailed Student's *t*‐test was used. For the scratch wound assay, Welch's unpaired two‐tailed *t*‐test was applied because equal variances were not assumed. For experiments involving two independent factors, two‐way analysis of variance (ANOVA) followed by Tukey's post hoc test was used. A *p*‐value < 0.05 was considered statistically significant. Statistical analyses were performed using GraphPad Prism (v.5.0; GraphPad Software, La Jolla, CA, USA).

## 3 Results

### 3.1 Chemical Characterization of CSE

HPLC analysis of the CSE showed a peak at a retention time corresponding to that of an authentic nicotine standard. Consistent with this finding, GC–MS library analysis tentatively assigned nicotine with a high reverse match factor (RMF 99.4%) and a relative chromatographic peak area of 49.28%. In addition to nicotine, three compounds associated with cigarette mainstream smoke chemistry were tentatively assigned: pyrazine, methyl‐ (methylpyrazine); 3‐furaldehyde, a furan‐based aldehyde structurally related to furfural; and 2‐cyclopenten‐1‐one, 2‐methyl‐, a methylated cyclopentenone derivative (Table S2).

Alkylpyrazines, including methylpyrazine, are abundant nitrogen‐containing heterocyclic compounds generated during tobacco combustion [41]. Furan‐based aldehydes, including furfural, are recognized pyrolysis products of tobacco constituents [42]. Cyclopentenone derivatives have also been identified by GC–MS in gas‐phase extracts of cigarette smoke in previous cell‐based studies [43]. Collectively, the HPLC and GC–MS findings support the presence of nicotine and characteristic combustion‐derived constituents in the CSE preparation used in this study.

### 3.2 Upregulation of NOTCH1 and TRPV4 in A549 Cells Following Chronic CSE Exposure

A549 cells chronically exposed to CSE for 4 weeks exhibited increased expression of NOTCH1 and TRPV4 (Figure 1A). Both mRNA and protein levels of NOTCH1 and TRPV4 were significantly elevated in A549 CSE cells compared with parental A549 cells (Figure 1B). These findings confirm increased NOTCH1 and TRPV4 expression in A549 cells following chronic CSE exposure.

**FIGURE 1:**
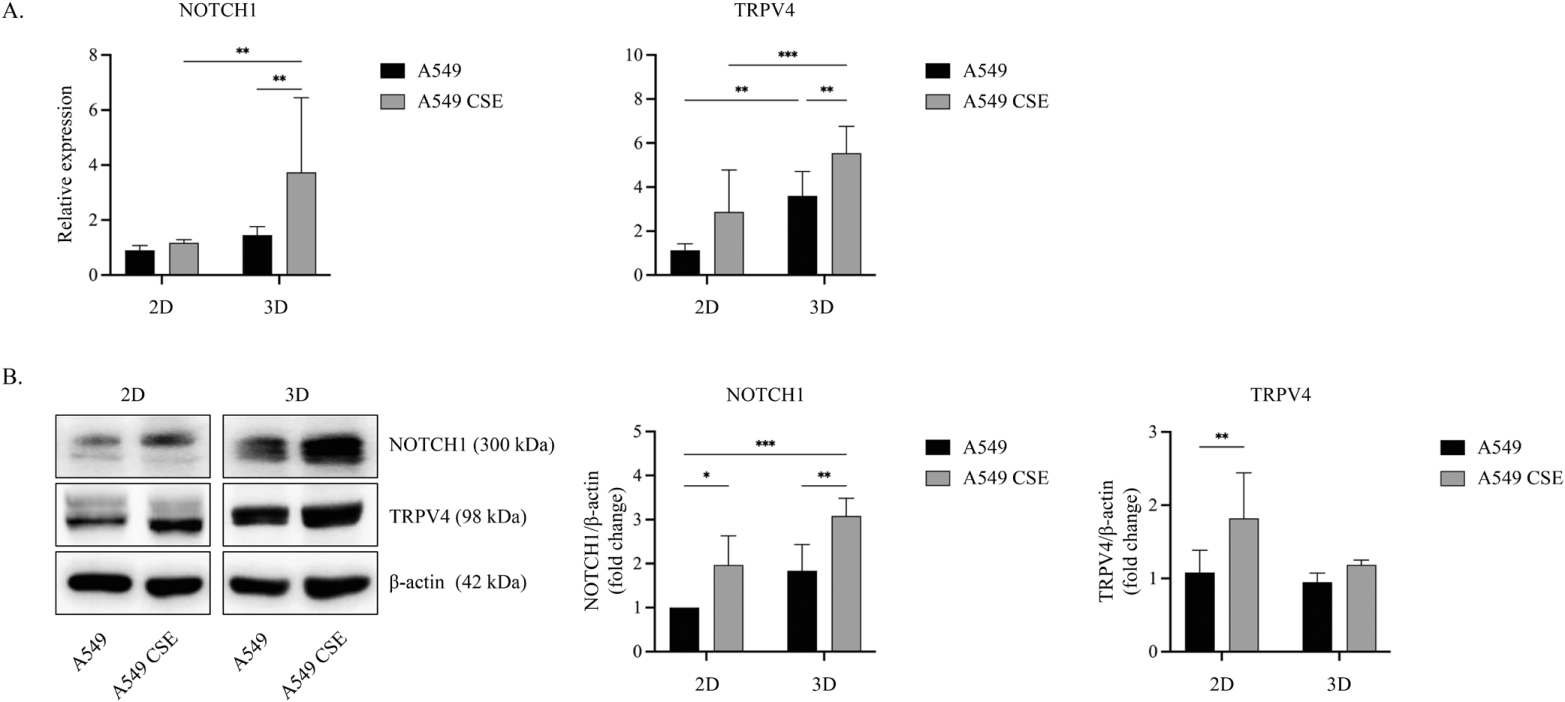
CSE increases NOTCH1 and TRPV4 expression in A549 cells under 2D and 3D culture conditions. (A) qRT‐PCR analysis of NOTCH1 and TRPV4 mRNA expression. (B) Representative western blot images and quantification of NOTCH1 and TRPV4 protein expression. β‐actin was used as a loading control. *p < 0.05, **p < 0.01, ***p < 0.001.

### 3.3 Enhanced Clonogenic, Migratory, and Invasive Phenotypes of A549 Cells Following Chronic CSE Exposure

Colony formation assays revealed that A549 CSE cells produced significantly more colonies than parental A549 cells (Figure 2A). MTT assays showed a trend toward increased cell viability in A549 CSE cells; however, the difference did not reach statistical significance (Figure 2B), suggesting that chronic CSE exposure may preferentially enhance clonogenic potential rather than short‐term metabolic activity. Scratch wound assays demonstrated that chronic CSE exposure significantly enhanced the migratory capacity of A549 cells at 24 h. A549 CSE cells exhibited substantially greater wound closure than parental A549 cells (82.95% ± 3.94% vs. 55.54% ± 2.03%, respectively; *p* = 0.0018, Welch's unpaired two‐tailed *t‐*test; *n* = 3 independent experiments) (Figure 2C). Similarly, Matrigel‐coated Transwell invasion assays demonstrated significantly greater invasive activity in A549 CSE cells than in parental A549 cells (Figure 2D). Collectively, these findings indicate that chronic CSE exposure promotes clonogenic, migratory, and invasive phenotypes in lung cancer cells.

**FIGURE 2:**
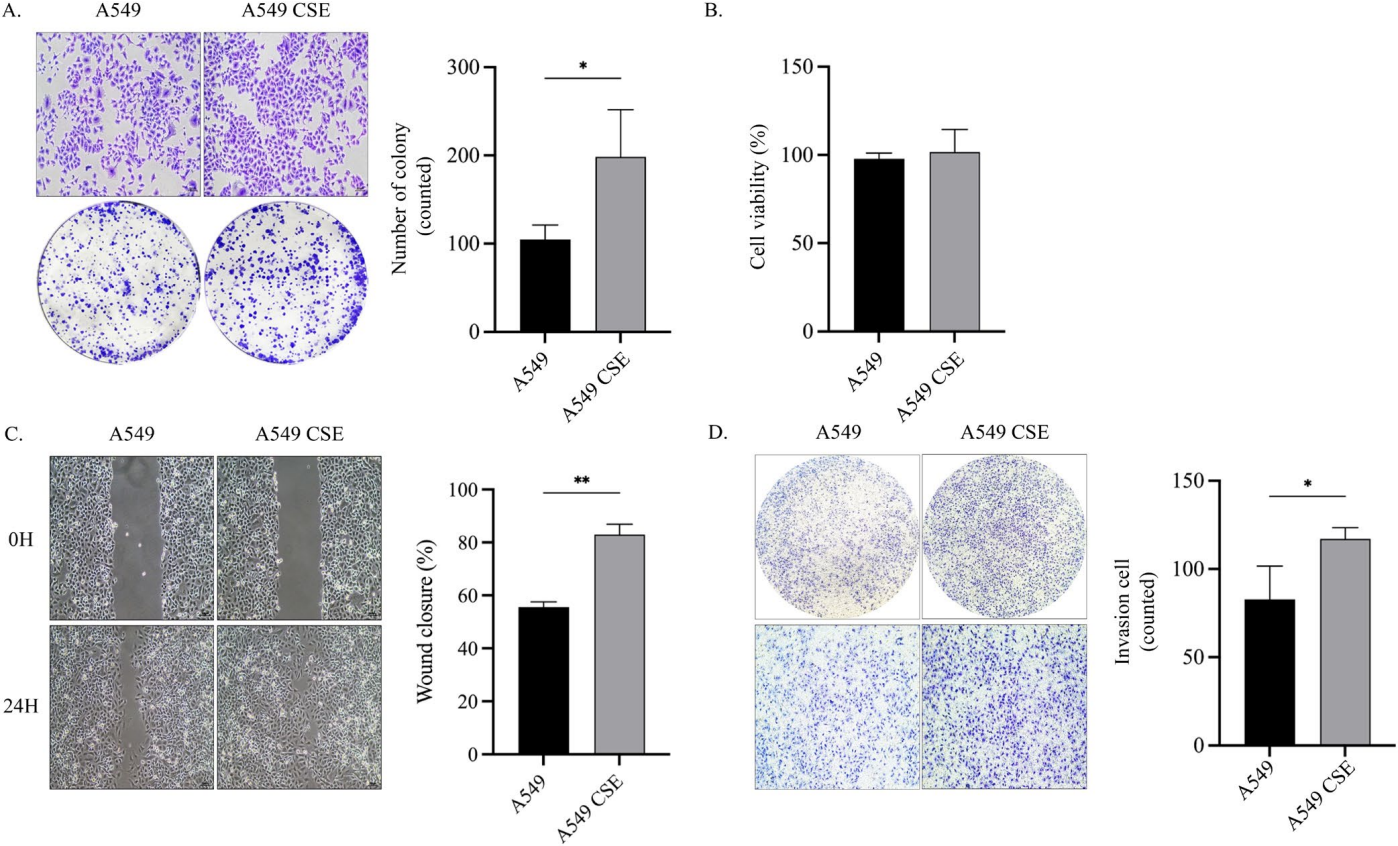
A549 CSE cells exhibit enhanced clonogenicity, migration, and invasion. (A) Representative images and quantification of colony formation in parental A549 cells and A549 CSE cells. (B) MTT assay comparing cell viability between parental A549 cells and A549 CSE cells. (C) Representative images of the scratch wound assay at 0 and 24 h and quantification of percent wound closure. Individual data points represent three independent biological experiments, and data are presented as the mean ± SD. Statistical significance was determined using Welch's unpaired two‐tailed *t*‐test. (D) Representative images and quantification of the Matrigel‐coated Transwell invasion assay. *p < 0.05, **p < 0.01.

### 3.4 Induction of Epithelial–Mesenchymal Transition (EMT)‐Associated Molecular Changes in A549 CSE Cells

The epithelial marker *E‐cadherin* showed no significant difference between A549 and A549 CSE cells (Figure 3). In contrast, mesenchymal markers, including N‐cadherin, Vimentin, and Snail, were significantly upregulated at both the mRNA (Figure 3A) and protein levels (Figure 3B) in A549 CSE cells. β‐catenin expression was significantly increased at the mRNA level, and protein expression was also elevated in A549 CSE cells, although the difference did not reach statistical significance. These findings suggest that chronic CSE exposure induces molecular changes associated with EMT in lung cancer cells.

**FIGURE 3:**
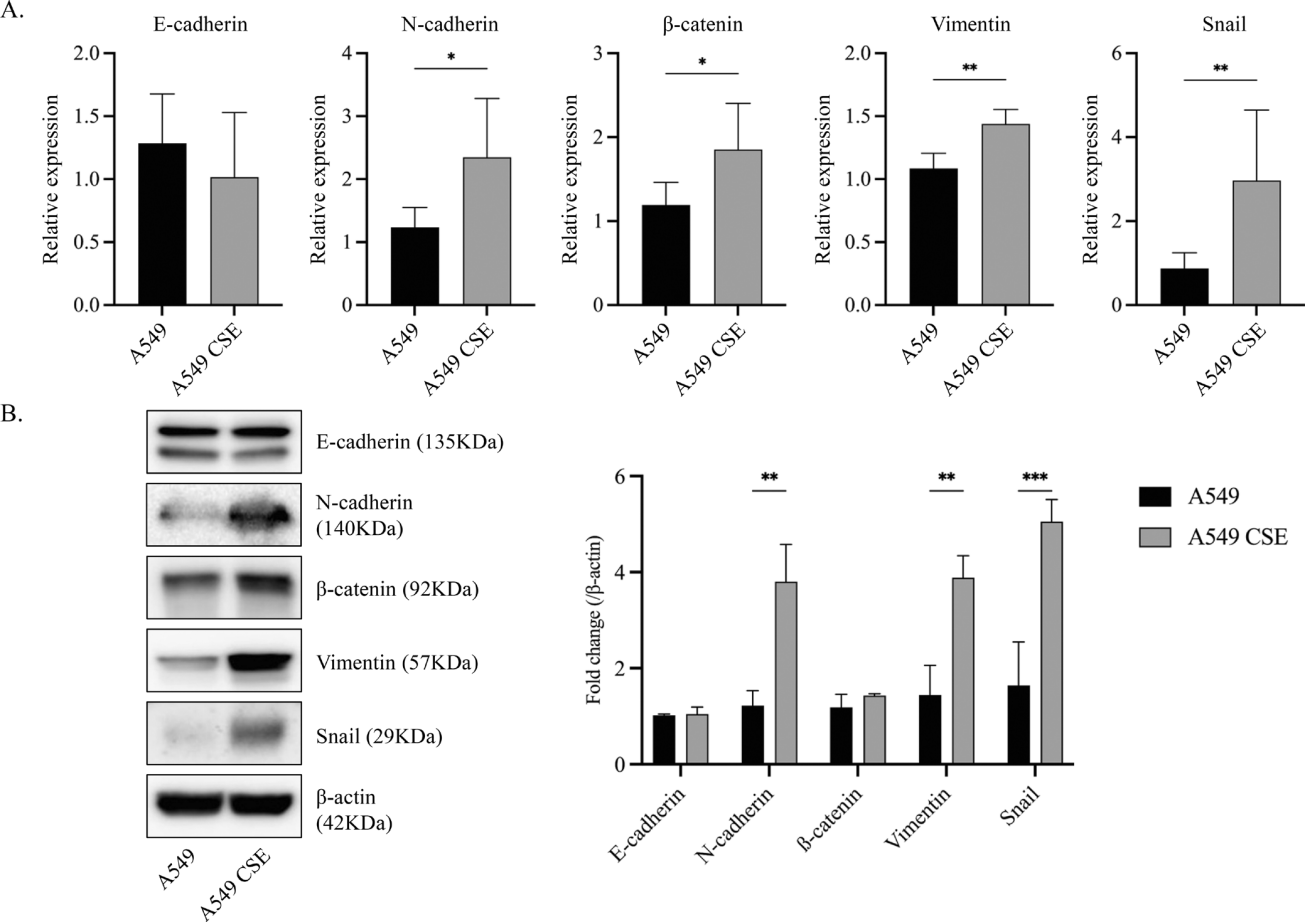
CSE induces EMT‐associated molecular changes in A549 cells. (A) qRT‐PCR analysis of E‐cadherin, N‐cadherin, β‐catenin, Vimentin, and Snail mRNA expression in parental A549 cells and A549 CSE cells. (B) Representative western blot images and quantification of E‐cadherin, N‐cadherin, β‐catenin, Vimentin, and Snail protein expression in parental A549 cells and A549 CSE cells. β‐actin was used as a loading control. *p < 0.05, **p < 0.01, ***p < 0.001.

### 3.5 Chronic CSE Exposure Enhances Tumor Growth in a Spheroid‐Based Xenograft Model

To evaluate tumorigenic potential in vivo, spheroids generated from A549 and A549 CSE cells were transplanted into nude mice to establish a spheroid‐induced lung cancer model (Figure 4A). Both A549 and A549 CSE cells successfully formed spheroids, and no significant difference in spheroid morphology was observed between the two groups (Figure 4B).

**FIGURE 4:**
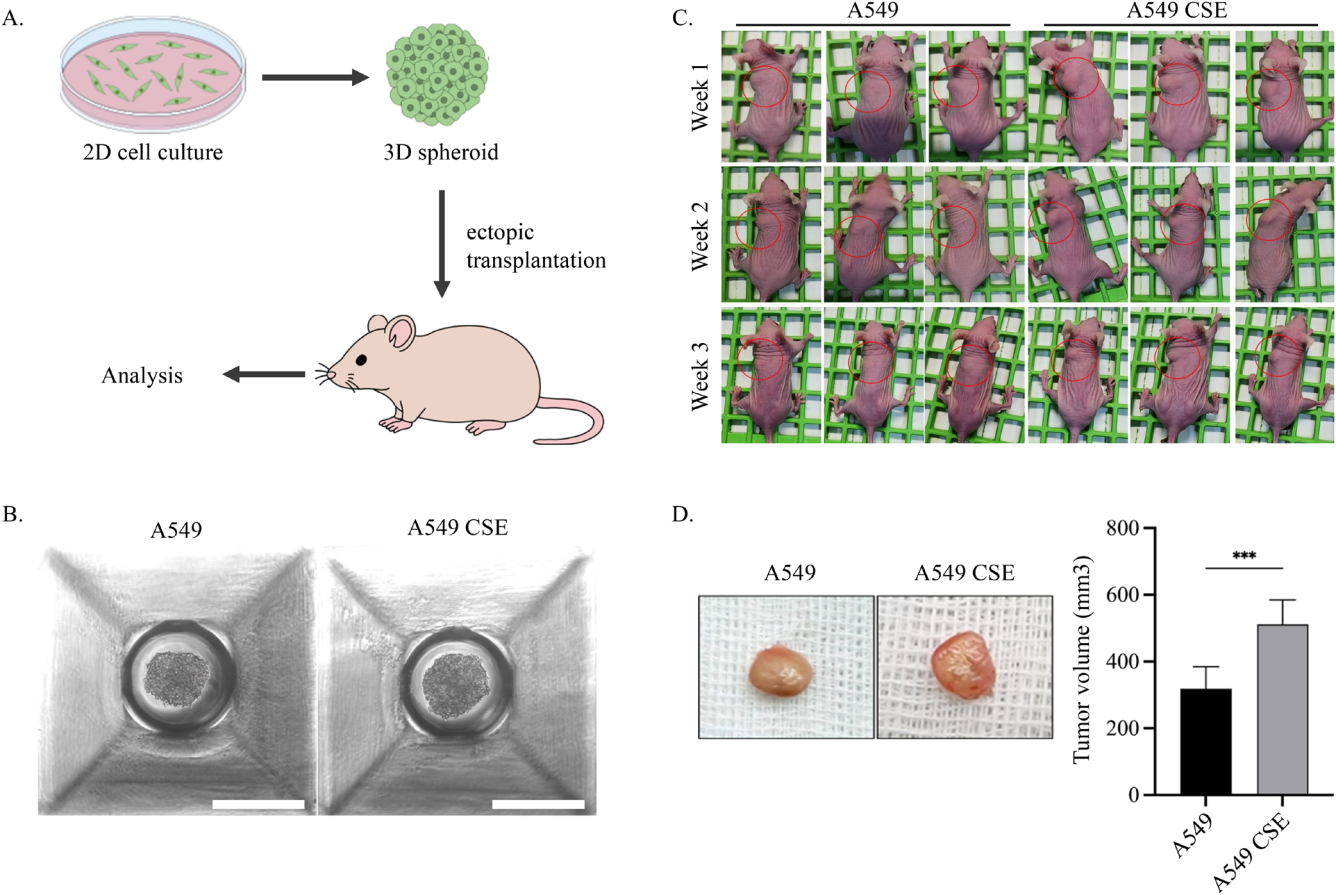
A549 CSE cells exhibit enhanced tumor growth in an in vivo xenograft model. (A) Schematic illustration of the in vivo xenograft model established by ectopic transplantation of tumor spheroids into a mouse model. (B) Representative images of tumor spheroids derived from parental A549 cells and A549 CSE cells prior to transplantation. Scale bar = 500 μm. (C) Representative images of mice bearing xenograft tumors over the indicated time course. Tumor regions are circled in red. (D) Representative images of excised tumors and quantification of tumor volume at the experimental endpoint. ***p < 0.001.

However, mice injected with A549 CSE spheroids developed significantly larger tumors than those injected with parental A549 spheroids (Figure 4C). Tumor volume in the A549 CSE group increased progressively and was significantly greater after 4 weeks.

Histological examination showed prominent mitotic figures and cellular atypia, along with reduced necrotic areas in tumors derived from A549 CSE cells compared with those derived from A549 cells (Figure 5A). Consistently, EMT‐associated proteins, including β‐catenin, N‐cadherin, and Vimentin, were markedly elevated in tumors derived from A549 CSE cells (Figure 5B,C).

**FIGURE 5:**
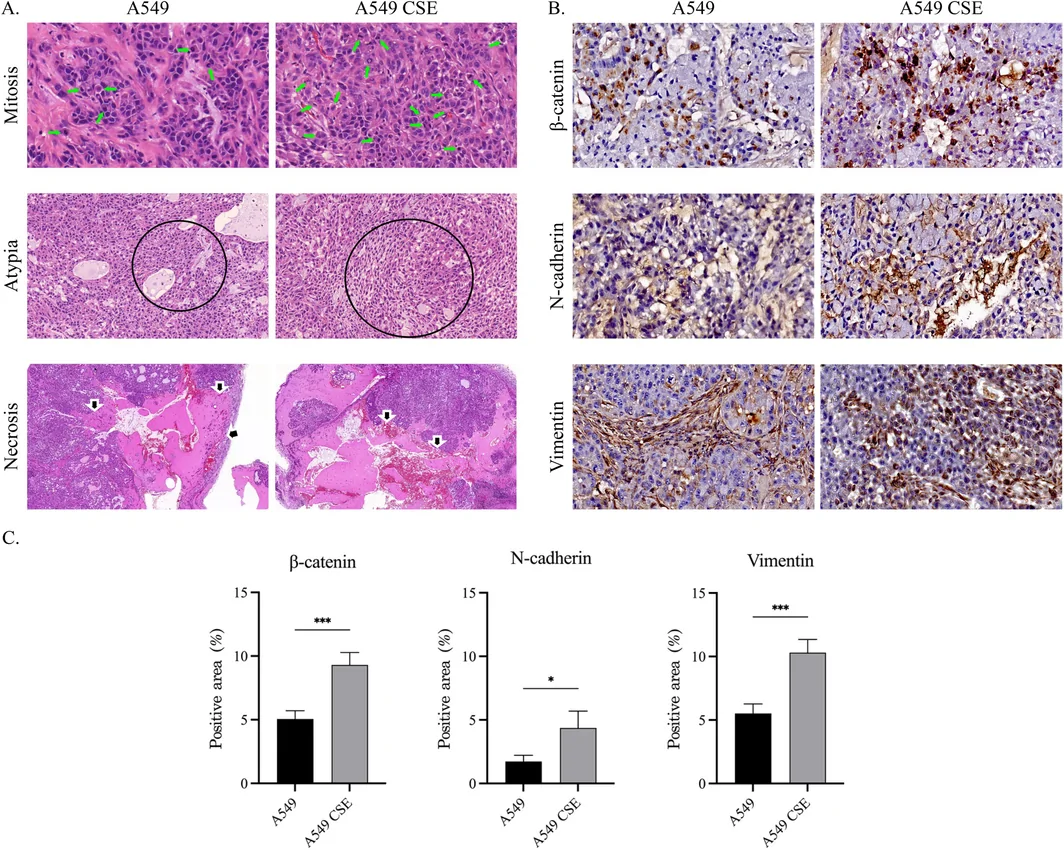
A549 CSE xenografts exhibit enhanced histopathological progression and EMT‐associated marker expression. (A) Representative hematoxylin and eosin‐stained sections of xenograft tumors derived from parental A549 cells and A549 CSE cells. (B, C) Representative immunohistochemical staining image and quantification of xenograft tumor sections for β‐catenin, N‐cadherin, and Vimentin. *p < 0.05, ***p < 0.001.

### 3.6 Increased Cell Motility of A549 CSE Cells in a Microfluidic 3D Model

To further evaluate cellular behavior in a physiologically relevant microenvironment, A549 and A549 CSE cells were cultured in a microfluidic chip‐based 3D system. A549 CSE cells exhibited markedly increased cell movement compared with parental A549 cells (Figure 6A).

**FIGURE 6:**
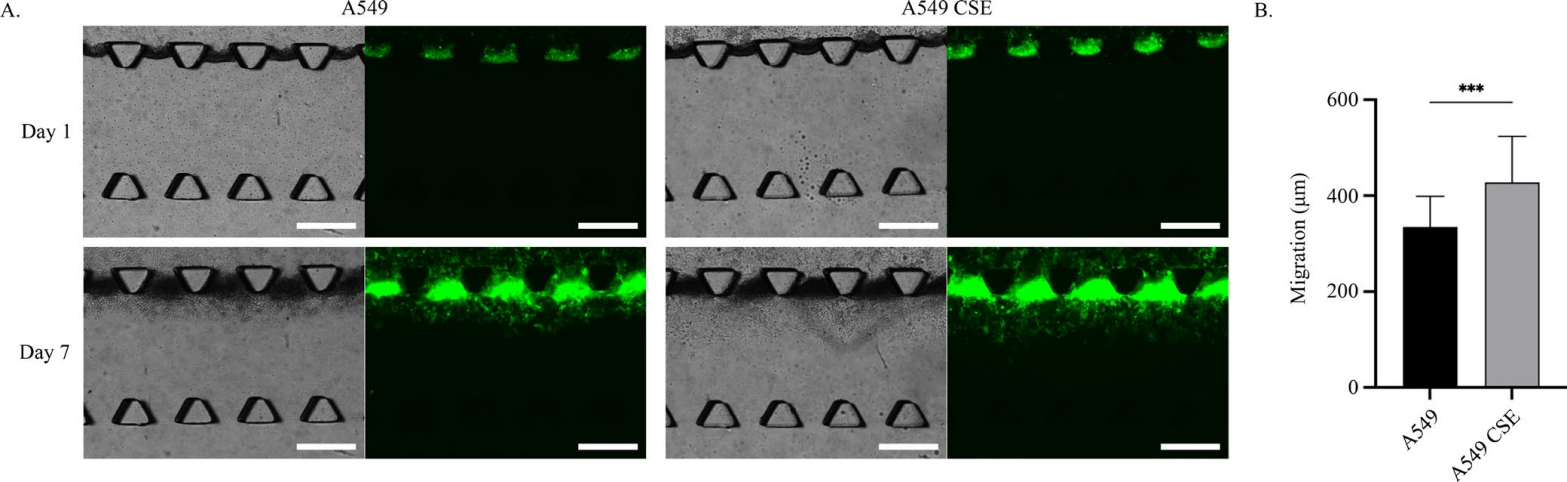
A549 CSE cells exhibit increased motility in a microfluidic 3D chip model. (A) Representative bright‐field and fluorescence images of parental A549 cells and A549 CSE cells cultured in a microfluidic 3D chip system on Day 1 and Day 7. Scale bar = 500 μm. (B) Quantification of migration distance in parental A549 cells and A549 CSE cells cultured in the microfluidic chip model. ***p < 0.001.

Quantitative analysis confirmed significantly greater cellular motility in A549 CSE cells (Figure 6B), supporting the enhanced invasive phenotype induced by chronic CSE exposure.

### 3.7 RNA‐Seq Analysis Reveals Transcriptional Reprogramming and Smoking‐Associated Mutational Signatures in CSE‐Exposed Lung Cancer Cells

RNA sequencing revealed substantial transcriptional alterations in A549 cells following 4 weeks of chronic CSE exposure (Figure 7A). Several genes associated with tumor progression and cellular stress responses were differentially expressed, including *TGFβR3, CCL5, KRT4, RAD51*, and *CDK2*.

**FIGURE 7:**
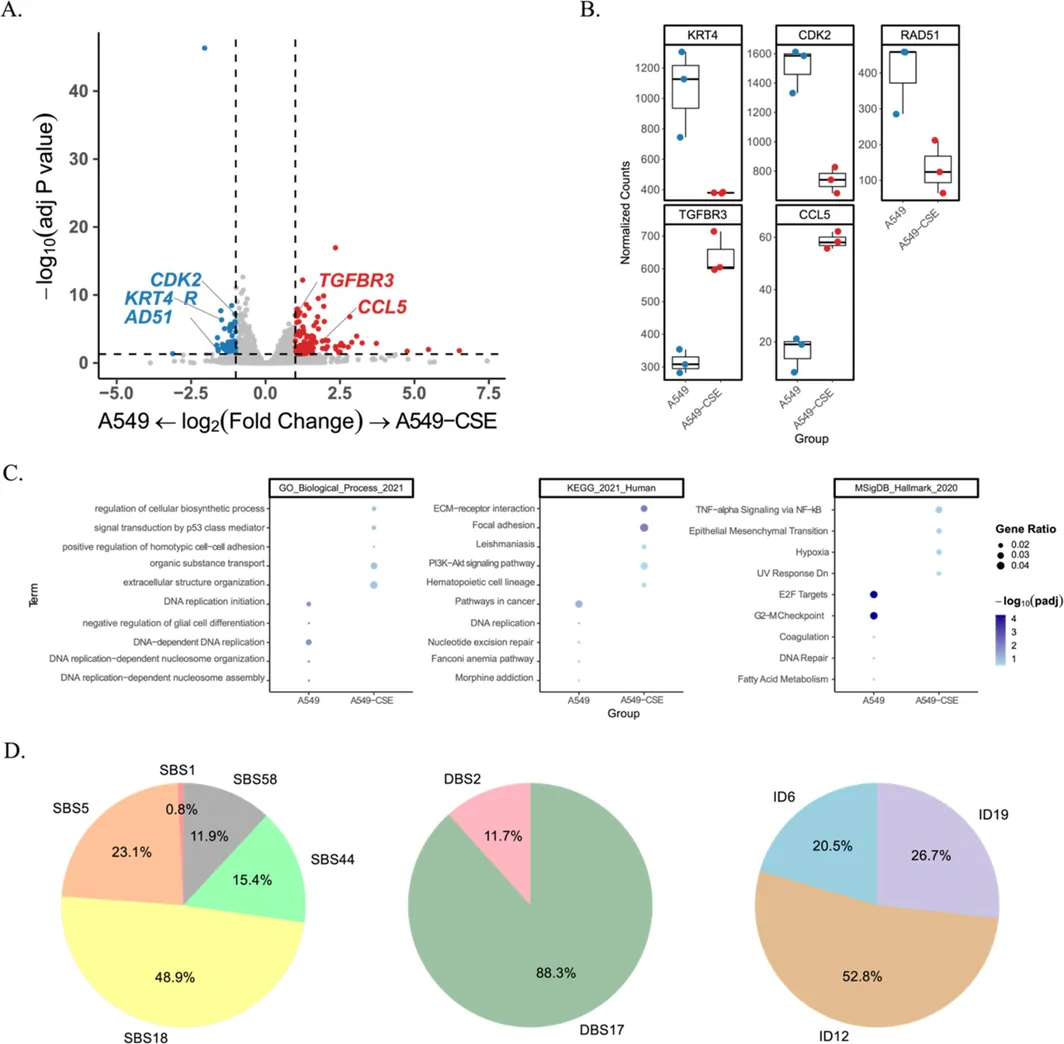
CSE‐induced transcriptional reprogramming and smoking‐associated mutational signatures in A549 cells. (A) Volcano plot of differential gene expression between parental A549 cells and A549 CSE cells after 4 weeks of chronic CSE exposure. Selected upregulated genes (*TGFβR3* and *CCL5*) and downregulated genes (*KRT4*, *CDK2*, and *RAD51*) are indicated. (B) Box plots showing normalized RNA‐seq expression counts of representative differentially expressed genes. (C) Pathway enrichment analysis of differentially expressed genes. (D) Whole‐genome sequencing‐based mutational signature analysis of A549 CSE cells, showing single base substitution (SBS), doublet base substitution (DBS), and small insertion/deletion (ID) signatures.

*TGFβR3* and *CCL5* were significantly upregulated, suggesting activation of inflammatory and tumor‐promoting signaling pathways. In contrast, *KRT4* and *CDK2* were downregulated, indicating alterations in epithelial characteristics and cell cycle regulation. Normalized expression counts further confirmed these differential expression patterns (Figure 7B).

Pathway enrichment analysis identified activation of several biological processes in response to chronic CSE exposure, including inflammatory signaling, EMT, and DNA damage response pathways (Figure 7C). In contrast, pathways associated with DNA replication initiation and DNA‐dependent DNA replication were significantly downregulated.

WGS further revealed smoking‐associated mutational signatures in CSE‐treated A549 cells. These included oxidative damage‐related signatures (SBS18), mismatch repair deficiency (SBS44), and mutational patterns associated with exposure to endogenous or exogenous mutagens, such as tobacco smoke and acetaldehyde (DBS2) (Figure 7D). Additionally, the ID6 signature, associated with defective homologous recombination‐mediated DNA repair, was also detected.

### 3.8 Validation of RNA‐Seq Findings by qRT‐PCR

To validate the RNA‐seq results, qRT‐PCR was performed for selected genes associated with tumor progression and DNA repair. Consistent with the transcriptomic analysis, *TGFβR3* and *CCL5* were significantly upregulated, whereas *RAD51* and *CDK2* were significantly downregulated in A549 CSE cells compared with parental A549 cells (Figure 8).

**FIGURE 8:**
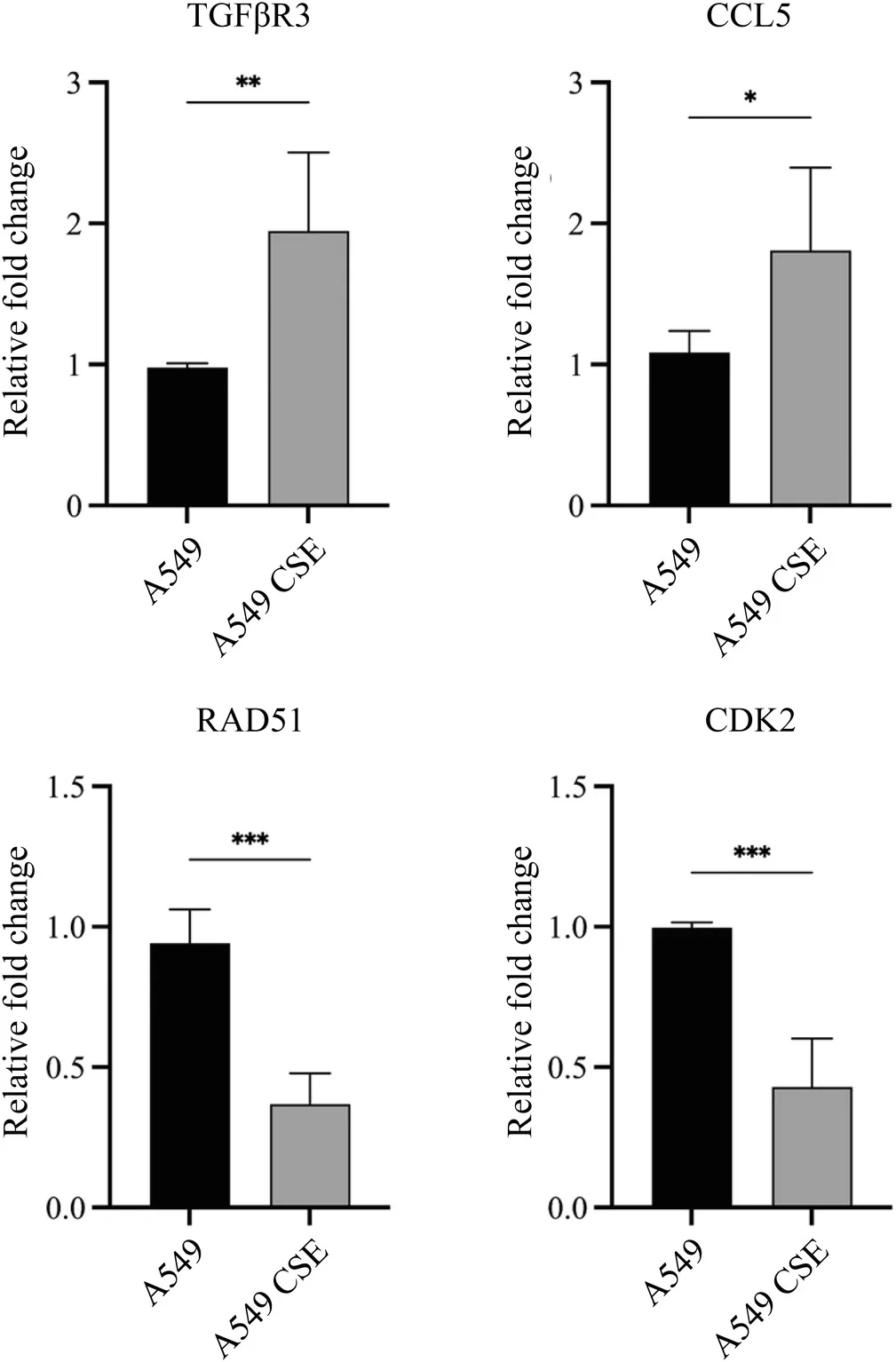
qRT‐PCR validation of RNA‐seq–identified differentially expressed genes in A549 CSE cells. These results confirm the reliability of the RNA‐seq data and further support that chronic CSE exposure induces transcriptional alterations associated with inflammation, epithelial plasticity, and impaired DNA repair pathways in lung cancer cells. *p < 0.05, **p < 0.01, ***p < 0.001.

## 4 Discussion

Cigarette smoking is the principal etiological factor for lung cancer and has long been recognized as the dominant environmental driver of lung carcinogenesis [1, 2, 3, 44]. Tobacco smoke contains numerous carcinogenic compounds capable of inducing DNA damage, mutagenesis, and genomic alterations during the early stages of tumor development [3, 5, 6, 45]. Although the role of smoking in tumor initiation has been extensively characterized, considerably less is known about how continued exposure to cigarette smoke influences the biological behavior of already established lung cancers. Clinical studies have nevertheless shown that persistent smoking after lung cancer diagnosis is associated with poorer survival outcomes and increased risk of recurrence [7, 8, 9, 10, 11]. These observations raise an important biological question: whether ongoing smoke exposure may actively promote tumor progression after malignant transformation has already occurred.

In the present study, we addressed this question by establishing a CSE exposure model in lung cancer cells and investigating its phenotypic and genomic consequences using multiple experimental platforms. A549 cells were repeatedly exposed to 1.5% CSE for 48 h during each 3‐day cycle over approximately 4 weeks, thereby modeling sustained and recurrent smoke‐associated toxic stress in already transformed lung cancer cells. By integrating conventional phenotypic assays, three‐dimensional culture systems, xenograft models, transcriptomic profiling, and WGS, our findings suggest that persistent exposure to cigarette smoke may accelerate tumor evolution in established lung cancer.

Our phenotypic analyses demonstrated that chronic CSE exposure enhances aggressive tumor behavior. Chronic exposure significantly increased colony‐forming capacity, scratch wound closure, and Transwell invasion, whereas the increase observed in the MTT assay did not reach statistical significance. These findings suggest that chronic CSE exposure preferentially enhances clonogenic, migratory, and invasive properties rather than short‐term metabolic activity. Chronic exposure increased the proliferative capacity, colony‐forming ability, and migratory activity of lung cancer cells. These functional alterations were accompanied by molecular changes consistent with EMT, including increased expression of N‐cadherin, Vimentin, β‐catenin, and Snail, while E‐cadherin expression remained relatively preserved, consistent with a partial EMT phenotype rather than a complete EMT [16]. Similar CSE‐associated EMT‐related changes have been reported in lung models [12, 16]. Recent studies have highlighted that partial EMT states represent an important mechanism underlying tumor plasticity and metastatic competence, enabling cancer cells to retain proliferative capacity while acquiring migratory properties [46, 47, 48]. In this context, our findings suggest that chronic cigarette smoke exposure may promote tumor cell plasticity associated with increased invasive potential.

The tumor‐promoting effects of chronic smoke exposure were further supported by our in vivo and microenvironment‐mimicking models. Xenograft tumors derived from CSE‐exposed spheroids exhibited significantly increased tumor growth and greater cellular atypia compared with tumors derived from parental cells. Furthermore, EMT‐associated proteins were elevated in tumors derived from CSE‐exposed cells, supporting the phenotypic alterations observed in vitro. Microfluidic chip‐based three‐dimensional cultures also revealed enhanced cellular motility under physiologically relevant conditions. These findings indicate that chronic cigarette smoke exposure promotes aggressive tumor behavior across multiple biological contexts, including conventional cell culture systems, microenvironment‐mimicking platforms, and in vivo tumor models.

Beyond these phenotypic alterations, our transcriptomic and genomic analyses revealed evidence of increased genomic instability, a key factor in tumor progression, following chronic CSE exposure [49]. RNA‐sequencing analysis demonstrated dysregulation of genes involved in cell‐cycle regulation, DNA replication, and inflammatory signaling. In particular, the downregulation of RAD51 suggests impaired homologous recombination‐mediated DNA repair, a mechanism known to contribute to genomic instability and tumor progression [50, 51]. Defects in DNA damage response pathways represent a fundamental driver of tumor evolution and therapeutic resistance in many cancers [52]. Consistent with this concept, pathway enrichment analysis in our study indicated suppression of DNA replication and cell‐cycle regulatory pathways following chronic smoke exposure.

WGS further supported the presence of smoke‐associated genomic alterations in chronically exposed cells. Mutational signature analysis revealed oxidative damage‐associated signatures (SBS18), mismatch repair‐related signatures (SBS44), and homologous recombination‐deficiency‐associated signatures (ID6). Large‐scale cancer genome studies have previously demonstrated that tobacco exposure is strongly associated with increased mutational burden and characteristic smoking‐related mutational signatures in lung cancer [20, 21, 22, 53, 54, 55]. Our findings extend these observations by suggesting that continued exposure to cigarette smoke may further reshape the genomic landscape of already transformed lung cancer cells.

Taken together, our results support a model in which persistent cigarette smoke exposure contributes not only to tumor initiation but also to the continued evolution of established lung cancers. By simultaneously promoting aggressive tumor phenotypes and destabilizing genomic integrity, chronic smoke exposure may create conditions that accelerate tumor progression and heterogeneity.

This perspective may also provide a biological explanation for clinical observations showing poorer outcomes among patients who continue smoking after lung cancer diagnosis [9, 10, 15, 16, 56, 57]. Continued exposure to cigarette smoke may therefore not merely reflect an epidemiological risk factor but may actively contribute to tumor progression by promoting genomic instability and phenotypic plasticity in cancer cells.

Several limitations should be considered. First, this study used a single established lung cancer cell line, which may not fully represent the heterogeneity of human lung cancers. Future studies using additional cell lines and patient‐derived models are therefore warranted. In addition, proliferation‐specific markers such as Ki‐67 were not evaluated; therefore, the observed increases in clonogenicity and xenograft growth should not be interpreted as direct evidence of enhanced mitotic activity. Future studies incorporating Ki‐67 and other proliferation‐specific markers will be required to validate this phenotype. Second, the vacuum pump‐based CSE generation system does not fully reproduce the controlled puffing and smoldering phases of standardized smoking regimens, potentially altering combustion temperature and smoke composition [30]. Although HPLC and GC–MS analyses supported the presence of nicotine and several combustion‐derived compounds [29, 30, 31], the analyses did not provide comprehensive quantitative chemical profiling. Moreover, filtration through a 0.22‐μm membrane likely reduced particulate‐phase and tar‐associated constituents. Thus, the observed effects should be interpreted as responses to the defined, filtered CSE preparation rather than to the complete composition of cigarette mainstream smoke. Future studies employing standardized smoke‐generation protocols and comprehensive chemical profiling would help validate these findings.

In conclusion, our study demonstrates that chronic cigarette smoke exposure promotes aggressive tumor phenotypes and genomic instability in lung cancer cells. By integrating phenotypic, transcriptomic, and genomic analyses across multiple experimental platforms, we provide evidence that persistent smoke exposure may accelerate tumor evolution in established lung cancer. These findings highlight the biological importance of smoking cessation even after lung cancer diagnosis and provide new insights into how continued environmental carcinogen exposure may shape tumor progression.

## Author Contributions

**Hyun Soo Kim:** writing – original draft, writing – review and editing, formal analysis, visualization, investigation, methodology. **Se‐Eun Choi:** writing – review and editing, writing – original draft, formal analysis, data curation, investigation. **In Kyoung Kim:** writing – original draft, writing – review and editing, visualization, formal analysis, conceptualization, methodology, investigation. **Seulbee Lee:** investigation. **Seo‐Hee You:** investigation. **Rachel Hyunkyung Kim:** investigation. **Chang Dong Yeo:** writing – review and editing, resources, supervision. **Jin‐A Kim:** writing – review and editing, supervision. **Seok Chung:** writing – review and editing, resources, supervision. **Sang Haak Lee:** writing – review and editing, resources. **Semin Lee:** writing – review and editing, resources, supervision. **Hye Joung Kim:** writing – review and editing, writing – original draft, conceptualization, methodology, project administration.

## Funding

This work was supported by National Research Foundation of Korea, NRF‐2022R1I1A1A01063972, RS‐2025‐00514943, RS‐2018‐NR031072, RS‐2023‐00225255, RS‐2023‐00261820, RS‐2024‐00466776; Ministry of Trade, Industry and Energy (MOTIE), Republic of Korea, 1415180884, 20012378; and Technology Innovation Program, RS‐2025‐02314555.

## Ethics Statement

All animal experiments were approved by the Institutional Animal Care and Use Committee of the Catholic University of Korea (CUMC‐2021‐0292‐06).

## Conflicts of Interest

The authors declare no conflicts of interest.

## Supporting information


**Table S1:** Chemical characterization of the CSE used for cell treatment.

**Table S2:** GC–MS library analysis of the cigarette smoke extract used for cell treatment.

## Data Availability

The datasets used and/or analyzed during the current study are available from the corresponding author on reasonable request.
