# Supplementary material for: Chronic Cigarette Smoke Extract Exposure Promotes Genomic and Phenotypic Reprogramming in Lung Cancer Cells
Source: Thorac Cancer. 2026 Oct 9;17(19):e70392. doi: 10.1111/1759-7714.70392 (PMC13652954; doi:10.1111/1759-7714.70392)
Supplement: Supplementary file 1 [file TCA-17-e70392-s001.docx]

**Supporting Information**

**Chronic cigarette smoke extract exposure promotes genomic and phenotypic reprogramming in lung cancer cells**

**Supplementary Table 2. GC–MS library analysis of the cigarette smoke extract used for cell treatment**

| **No.** | **RT (min)** | **Compound (GC-MS Library ID)** | **CAS No.** | **RMF (%)** | **Area (%)** |
| --- | --- | --- | --- | --- | --- |
| 1 | 3.292 | N,N,O-Triacetylhydroxylamine | 17720-63-7 | 94.4 | 10.59 |
| 2 | 4.578 | Picolinamide | 1452-77-3 | 78.2 | 2.70 |
| 3 | 6.976 | Pyrazine, methyl- (methylpyrazine) | 109-08-0 | 95.1 | 2.04 |
| 4 | 7.171 | 3-Furaldehyde (furan-based aldehyde, structurally related to furfural) | 498-60-2 | 90.4 | 6.79 |
| 5 | 9.045 | 2-Cyclopenten-1-one, 2-methyl- (methylated cyclopentenone) | 1120-73-6 | 93.6 | 2.52 |
| 6 | 16.635 | Glycerol 1,2-diacetate | 102-62-5 | 98.3 | 26.08 |
| 7 | 16.878 | Nicotine | 54-11-5 | 99.4 | 49.28 |
