# Supplementary material for: Chronic Cigarette Smoke Extract Exposure Promotes Genomic and Phenotypic Reprogramming in Lung Cancer Cells
Source: Thorac Cancer. 2026 Oct 9;17(19):e70392. doi: 10.1111/1759-7714.70392 (PMC13652954; doi:10.1111/1759-7714.70392)
Supplement: Supplementary file 2 [file TCA-17-e70392-s002.docx]

**Supporting Information**

**Chronic cigarette smoke extract exposure promotes genomic and phenotypic reprogramming in lung cancer cells**

**Supplementary Table 1. Chemical characterization of the CSE used for cell treatment**

| **Analyte** | **Result** |
| --- | --- |
| Nicotine | Peak detected in the CSE sample at a retention time identical to that of the authentic nicotine standard, confirming the presence of nicotine. |
